# An Accidental Intestinal Myiasis Caused by *Cochliomyia macellaria*

**DOI:** 10.1155/2021/6678411

**Published:** 2021-02-16

**Authors:** P. P. Jayawardana, T. C. Yahathugoda

**Affiliations:** ^1^Department of Pediatrics, Faculty of Medicine, University of Ruhuna, Matara, Sri Lanka; ^2^Department of Parasitology Faculty of Medicine, University of Ruhuna, Matara, Sri Lanka

## Abstract

Intestinal myiasis is recognized as pseudomyiasis or accidental myiasis caused by dipteran fly larvae transmitted to humans via contaminated food or water. A case of intestinal myiasis acquired via contaminated food is reported in this case study. The patient is a 4-year-old boy who had frequent episodes of crampy abdominal pain and diarrhoea and the passage of many live worms at each time. As the child had the habit of eating ripe guava from his garden, the infection source was suggested as ripe guava, and the possibility was explored. All larvae collected from faeces and fruit were morphologically similar, and it has been identified as *Cochliomyia macellaria*. The treatment with several antihelmintics failed, and the recovery was achieved with a simple measure of abstinence from eating guava that came from his garden.

## 1. Introduction

Infestation of live human or any vertebrate host with dipteran fly larvae is called myiasis [1]. Human myiasis can manifest as cutaneous myiasis, wound myiasis, oracular myiasis, and nasopharyngeal myiasis in common; however, anal myiasis, body cavity myiasis, aural myiasis, and intestinal myiasis are also reported [2]. Myiasis is classified into facultative, obligatory, and accidental categories depending on their survival on dead or live tissue. Fly larvae that requires living tissue for their survival cause obligatory myiasis. In contrast, those who live on dead or necrotic tissue cause facultative myiasis. The fly larvae that are accidentally ingested or deposited on living tissue cause accidental myiasis [1].

The first report on human myiasis in Sri Lanka was published in 1954 [3]. Only a limited number of indigenous cases were published to date [4–7]. Cutaneous myiasis was the commonest form, and *Chrysomya bezziana* (Old world screwworm fly) and *C. megacephala* were documented fly larvae [3, 6, 7]. *Cordylobia anthropophaga* (tumbu fly) was also reported in Sri Lanka as imported cases of cutaneous myiasis [8, 9]. Other common myiasis-causing agents such as *Cochliomyia hominivorax* (New world screwworm fly) and *Sarcophaga* spp. (flesh fly or sarcophagids) are not documented in Sri Lanka [7, 10].

People who have low socioeconomic conditions and live in a poor hygienic environment are at a risk of developing myiasis [10]. Intestinal myiasis or pseudomyiasis or accidental myiasis caused by ingestion of dipteran fly eggs via contaminated food or water is commonly seen in such communities [11]. However, fly larval infestation is most often self-limiting and does not result in any serious complications, which is a major cause of underreporting of human myiasis throughout the world. Hitherto, a case of intestinal myiasis is not reported in Sri Lanka. Diagnosis of human myiasis is usually missed by physicians due to lack of suspicion and have little idea of the specific clinical features [1]. Therefore, this case report aims to improve physicians' knowledge of intestinal myiasis by considering intestinal myiasis in the differential diagnosis list when assessing acute and chronic abdominal pains, especially in children.

## 2. Case Presentation

A 4-year-old previously healthy boy had several (seven) episodes of colicky abdominal pain and loose stools over ten months. These episodes were never associated with fever but have had loss of appetite and infrequent vomiting. He had perianal discomfort both in the day and night. His mother had observed the passage of live worms with faeces on all these occasions, and she emphasized that it was more than 500 in number. During this period, no one else in the family had an illness similar to food poisoning, gastroenteritis, or worm infestation.

He was first seen by a general practitioner and treated for a common intestinal helminthiasis with mebendazole 100 mg twice daily for three days without obtaining laboratory evidence. After that, the child had been seen five times by general practitioners before the first author saw him. Stool investigations were done on two occasions and were reported as normal. It was not easy to get the treatment history due to retrograde exploration. The child had been treated with various anthelmintics and antibiotics. His mother could recall that he had been given both anthelmintics and antibiotics in a combination in few occasions. He had been treated with mebendazole 100 mg twice daily for three days in two occasions, albendazole 400 mg single dose in two occasions, and once treated by two tablets of pyrantel pamoate (250 mg). Whenever those episodes were predicted as food poisoning or infective gastroenteritis, the child had received either cephalexin or metronidazole along with zinc and probiotics for 3–5 days. Usually, the child became symptom-free in 6-7 days regardless of the treatment modality, but the reappearance of the symptoms in few weeks were inevitable.

The first author has seen the child in his sixth episode. The child complained of a gush of offensive loose stools with worms and abdominal cramps. A sample of stool has been sent for amoeba, ova, and cysts but became negative. Fecal culture was negative for bacteria. However, the child was treated empirically for helminthic infection using 400 mg albendazole single dose and repeated the same dose of treatment after a week. All family members were treated with the same regimen and instructed to follow hygienic measures. The child was brought to the same clinic in few weeks due to reappearance of similar symptoms. This occasion, the faeces were frothy and greenish. The parents have taken all possible hygienic measures to prevent the recurrences by introducing regular hand washing, cutting nails short etc. However, it did not prevent him getting recurrences.

**Figure 1:**
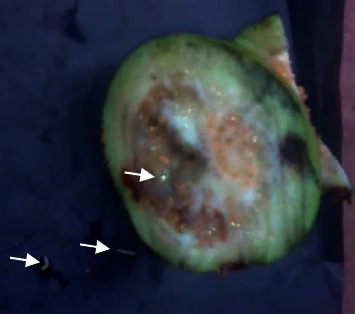
Guava fruit with fly larvae.

He was from a semiurban area. In his garden, there were many guava trees bearing fruits. He had the habit of eating ripe guava. Upon the authors' request, the parents collected a fecal sample and guava fruit with larvae (Figure 1). The parents succeeded in taking video-clips of live worms on faeces and fruits.

The second author examined all samples at the Department of Parasitology, Faculty of Medicine, University of Ruhuna. Live specimens were found in faeces and fruits. Faeces was examined using saline, iodine wet mounts, and Kato-Katz. It was negative for amoeba, ova, and cysts.

Live wormy specimens were submerged in hot water (∼95°C) for 30 seconds and then in 95% ethanol before examining them under a dissecting microscope. The average length of larvae was 7 mm.

### 2.1. Microscopic Identification of Larvae

**Figure 2:**
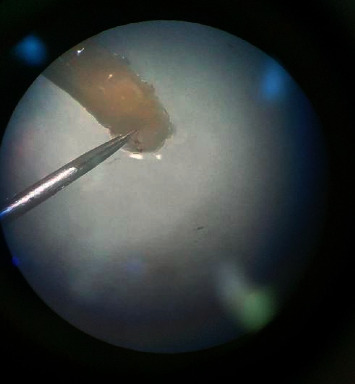
Posterior spiracles (before dissect).

**Figure 3:**
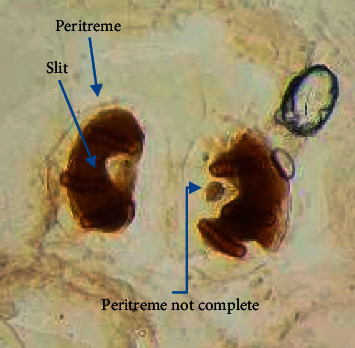
Posterior spiracles (after dissect).

**Figure 4:**
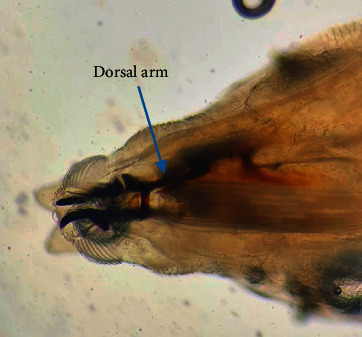
Arms of cephaloskeleton.

Pictorial key of Centers for Disease Control and Prevention, USA, was referred for larval identification (http://www.cdc.gov). All specimens collected did not have a definite, hard, sclerotized head capsule. The body was smooth and had short spines but no long lateral processes. Posterior spiracles were not on peg-like tubercles (Figure 2). Body was tapered posteriorly but did not extend into a tail-like process. Dissected posterior spiracle showed peritreme with three distinct slits which were positioned straight (Figure 3). The dorsal arm of cephaloskeleton is longer than the ventral arm and posterior spiracles with incomplete peritreme (Figures 3 and 4).

**Figure 5:**
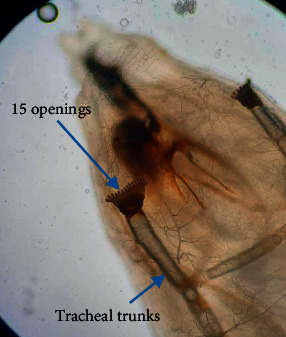
Prothoracic spiracles.

Spiracle slits point towards opening in peritreme (Figure 3). Prothoracic spiracles had 15 openings (Figure 5). Posterior spiracle button is indistinct, and wall of slits had swellings (Figure 3). Tracheal trunks were not pigmented (Figure 5). Larvae have been identified as *Cochliomyia macellaria.* All larvae collected from faeces and fruit were morphologically similar.

The parents were instructed to avoid possible exposure to eating fruits (guava) from their garden. The child was reviewed in 3 and 6 months. He was symptom-free, and larvae have ceased appearing in the faeces.

## 3. Discussion

A careful evaluation of fly larvae collected from the child's faeces and the fruit revealed that the both larval groups were morphologically similar, and the key described by the Centers for Disease Control and Prevention, USA (http://www.cdc.gov), identified the larvae as *Cochliomyia macellaria* (secondary screwworm). Intestinal myiasis caused by *Cochliomyia macellaria* (secondary screwworm) is new to world literature. Myiasis due to *Cochliomyia hominivorax (*New world screwworm) is well documented. *Cochliomyia hominivorax* is an obligatory parasite causing intestinal [12], auricular [13], oral [14], nasal [15], and wound [16] myiasis. *Cochliomyia macellaria* is a facultative parasite that infests on wound or necrotic tissue of man and livestock. Like *C. hominivorax*, *C. macellaria* does not feed on living tissues [17]. Livestock industry experiences huge economic losses due to parasitism (myiasis) and disease transmission caused by *C. macellaria* maggots. Different salmonella types, swine flu, and botulism in birds are transmitted by maggots of *C. macellaria* [17].

All reported cases of intestinal myiasis have caused only by facultative or accidental fly larvae, whereas obligatory gut parasites of animals have never been encountered in human gut [18].

Over 50 species of fly larvae mainly belonging to families Muscidae, Calliphoridae, and Sacrophagidae have been reported from cases of intestinal myiasis. The clinical presentations of enteric myiasis reported so far and their site of infestation and species identified are summarized in Table 1.

The child has had more than three episodes of abdominal pain within three months; therefore, the presentation can be considered as recurrent abdominal pain (RAP) in children [39]. Diagnosis of RAP depends on the clear history provided by the parent and the child. The common causes of RAP such as functional abdominal pain, constipation, irritable bowel, and peptic ulcers could be diagnosed clinically by taking a careful history and through physical examination [40]. Intestinal infections due to bacteria, protozoa, and helminths can be diagnosed by a direct stool examination and culture. RAP in Sri Lanka is most considered as functional abdominal pain (nonorganic) where associated symptoms such as fever, vomiting, and microorganism or blood in stool are not seen [39, 41]. Here, in this case, the child has experienced several episodes of vomiting, diarrhoea, and perianal discomfort other than the vague abdominal pain. Therefore, an organic cause was considered for the RAP, and the child was investigated with stool AOC and culture. Since none of the investigation became positive, an empiric cause of medical management was considered because it has greater value than multiple exclusionary investigations [40].

Intestinal myiasis is also considered as an organic cause for RAP [39]. Simple elimination of eating guava has improved the child's clinical condition. Therefore, we can assume that the symptoms mentioned above were due to ‘maggots' infestation, but it is an incontestable proof. Offensive greenish stools associated with colicky abdominal pain and vomiting may have been triggered by the inflammation caused by fly maggots [16]. Similar offensive breath and vomit has been reported due to gastric myiasis [34], (Table 1). Consumption of overripe or rotten fruits such as banana [32] and peach [33] has triggered intestinal myiasis (Table 1). Confirmation of transmission route by demonstrating the same species of fly larvae in guava fruit is a remarkable achievement. For the first time in Sri Lanka, such transmission route is established and for the first time in the world literature, guava as the fruit to transport fly eggs larvae. Studies conducted in Sri Lanka have documented *Megaselia scalaris* in ripe bananas; however, intestinal myiasis caused by them has not been reported [42].

There is no specific treatment for intestinal myiasis [18]. Anti-inflammatory and antiperistaltic agents will reduce the symptoms, whereas broad-spectrum anthelmintics and antibiotics will not be helpful. Therefore, conducting continuous education and communications programmes to improve the knowledge of prevention and precautions against intestinal myiasis for physicians and public health officials is needed. Success will be based on behavioral modification such as abstain from consuming uncovered food, which has access to fly, and overripe or rotten fruits and abstain from defecating in the open. We want to emphasize that clinicians must find species and transmission route before embarking on the therapy. The only successful treatment for enteric myiasis was colonic irrigation using oral polyethylene glycol (PEG) [18]. Ivermectin was tried with no significant effect [18].

## 4. Conclusion

Though the intestinal myiasis does not cause serious complication, the morbidity is substantial. Therefore, a great deal of suspicion and exploring a history of passage of worms will diagnose intestinal myiasis. Identification of the species and the route of transmission is of profound importance to eliminate the disease.

## Figures and Tables

**Table 1:** Clinical presentation, site of infestation, and the species of fly larvae in reported cases of enteric myiasis.

Clinical details	Affected area	Species	Reference
Seven cases were reported with passage of worms from 3 months to 6 years, vague abdominal discomfort, diarrhoea, and anaemia	Intestine	*Sarcophaga haemorrhoidalis* (4)	[18]
		*Sarcophaga* spp. (1)	
		*Magaselia* spp. (1)	
		*Muscina stabulans* (1)	

Comorbidity with intestinal myiasis and helminthic infection	Intestine	*Sarcophaga haemorrhoidalis*	[19]
Passage of live worms in stool	Intestine	*Sarcophaga* spp.	[20]
Comorbidity with intestinal myiasis and giardiasis	Intestine	*Sarcophaga* spp.	[21]

Three cases were presented: one case had comorbidity with *Salmonella enteritis*, and two other cases had asymptomatic passage of worms	Intestine	*Sarcophaga crassipalpis* (carrier of *Salmonella*) (1)	[22]
		*Sarcophaga peregrina* (1)	
		*Hermetia illucens* (1)	

Presented with vague abdominal pain and irritable bowel	Intestine	*Muscina stabulans*	[23],
Passage of worms in stool	Intestine	*Megaselia scalaris*	[24, 25]
Two cases were reported: passage of worms in stool	Intestine	*Musca domestica*	[26]
Passage of worms in stool	Intestine	*Eristalis Tenax*	[27–30]
An irritable child was passing blood and mucous diarrhoea	Intestine	*Sarcophaga peregrina*	[31]
Passage of worms in stool after consuming overripe banana	Intestine	*Muscina stabulans*	[32]
Passage of worms in stool after consuming overripe decaying peaches	Intestine	*Eristalis tenax*	[33]

Four cases were presented: three had offensive hematemesis and minute moving worms in the vomitus, and the other one had symptomatic passage of worms in stool	Gastric (3)	*Sarcophaga* spp. (2)	[34]
	Intestine (1)	*Oestrus* spp. (2)	

Passage of worms in stool	Intestinal	*Fannia canicularis*	[35]
Passage of worms in stool	Intestinal	*Hermetia illucens*	[36, 37]
Passage of worms in stool	Intestinal	*Phaenicia cuprina*	[38]

## Data Availability

The data used to support the finding of this study are included within the article.
